# The EHA Research Roadmap: Blood Coagulation and Hemostatic Disorders

**DOI:** 10.1097/HS9.0000000000000643

**Published:** 2021-09-10

**Authors:** Sabine Eichinger, Pierre E. Morange, Marco Cattaneo, Mathilde Fretigny, Antoine Rauch, Astrid van Hylckama Vlieg, David-Alexandre Trégouët, Wolfram Ruf, Marcel Levi, José A. Páramo, Tom van der Poll, Paul A. Kyrle, Isabella Garagiola, Flora Peyvandi

**Affiliations:** 1 Department of Medicine I, Division of Hematology and Hemostasis, Medical University of Vienna, Austria; 2 C2VN, INRAE, INSERM Aix-Marseille University, France; 3 Università degli Studi di Milano, Italy; 4 Hospices Civils de Lyon, France; 5 University of Lille, Inserm, CHU Lille, Institut Pasteur de Lille, France; 6 Department of Clinical Epidemiology, Leiden University Medical Center, The Netherlands; 7 University Bordeaux, INSERM UMR 1219, Bordeaux Population Health Research Center, France; 8 Center for Thrombosis and Hemostasis, Johannes Gutenberg University Medical Center Mainz, Mainz, Germany; 9 Amsterdam University Medical Centers, Department of Vascular Medicine, University of Amsterdam, The Netherlands; 10 Department of Medicine, University College London Hospitals, United Kingdom; 11 Clínica Universidad de Navarra, Pamplona, Spain; 12 Amsterdam University Medical Centers, Division of Infectious Diseases & Center of Experimental Molecular Medicine, University of Amsterdam, The Netherlands; 13 Fondazione IRCCS Ca’Granda Ospedale Maggiore Policlinico, Angelo Bianchi Bonomi Hemophilia and Thrombosis Center, Department of Pathophysiology and Transplantation, Università degli Studi di Milano, Italy

*In 2016, the European Hematology Association (EHA) published the EHA Roadmap for European Hematology Research* ^1^ *aiming to highlight achievements in the diagnostics and treatment of blood disorders, and to better inform European policy makers and other stakeholders about the urgent clinical and scientific needs and priorities in the field of hematology. Each section was coordinated by 1–2 section editors who were leading international experts in the field. In the 5 years that have followed, advances in the field of hematology have been plentiful. As such, EHA is pleased to present an updated Research Roadmap, now including 11 sections, each of which will be published separately. The updated EHA Research Roadmap identifies the most urgent priorities in hematology research and clinical science, therefore supporting a more informed, focused, and ideally funded future for European hematology research. The 11 EHA Research Roadmap sections include Normal Hematopoiesis; Malignant Lymphoid Diseases; Malignant Myeloid Diseases; Anemias and Related Diseases; Platelet Disorders; Blood Coagulation and Hemostatic Disorders; Transfusion Medicine; Infections in Hematology; Hematopoietic Stem Cell Transplantation; CAR-T and Other Cell-based Immune Therapies; and Gene Therapy.*

Thrombotic and bleeding disorders are global disease burdens with considerable morbidity and high mortality.^2^ Estimates for the European Union (EU) suggested an annual death toll of 500,000 venous thrombosis (VT)-related deaths with a short-term mortality rate comparable to ischemic stroke.^3,4^ On another scale, about 1 in 300 persons is affected by an inherited bleeding disorder. Uncontrolled bleeding is a major healthcare issue not only among these patients but also among patients with an acquired bleeding disorder.

Following-up on the previous addition of the EHA Roadmap,^1^ the following chapters will give you an overview and update on important developments and multidisciplinary aspects of hemostasis and thrombosis. Owing to the SARS-CoV-2 pandemic, we specifically focus on infections and coagulopathy. We will describe major needs and cutting-edge questions in basic and clinical research in this topic providing views on future developments.

## Genomics in hemostasis

### Introduction

Inherited disorders of the hemostatic system can be divided into those that increase the risk of bleeding and those that increase the risk of thrombosis.

#### Thrombosis

Venous thromboembolism (VTE) has a high heritability which is estimated around 40%.^5^ The spectrum of genetic factors contributing to VTE susceptibility ranges from common variants associated with low-to-moderate effects (eg, FV Leiden and FII G20210A) to private mutations segregating within families associated with very high risk of disease (such as private mutations responsible for inherited deficiency in coagulation inhibitors).

#### Bleeding

In 2020, the molecular diagnosis was recommended for all hemophilia patients regardless of the clinical phenotype. However, in about 1%–5% of hemophilia cases and in up to 35% of patients with Von Willebrand disease (VWD) type 1,^6,7^ no disease-causing variant could be found using conventional molecular diagnosis. Mutations in >50 genes have been associated with inherited defects of platelet biology and strong clinical heterogeneity.

### European research contributions

#### Thrombosis

As part of the INVENT consortium to which European partners contribute, a new meta-analysis of Genome Wide Association Study (GWAS) cohorts has been conducted and identified 17 susceptibility loci to VTE.^8^ This study capitalized on 30,234 cases and 172,122 controls with genome-wide genotype data, including the UK biobank. This study adds several loci to the ones linked to coagulation—all involved directly or indirectly in platelet or erythrocyte biology or inflammation. Most identified common genetic variants are weak risk factors, that is, those with relative risks between 1.0 and 1.5 in the general population. How this information can be used in the clinic remains to be better characterized. Beyond common variants and given the evolution of molecular technologies and the widespread use of next-generation sequencing (NGS) at low cost, it becomes possible to identify very rare disease mutations that could even lie outside the classical coagulation cascade.^9^

#### Bleeding

New technologies were continuously incorporated into European laboratories leading to the progressive reduction of unexplained hemophilia-A (HA). For example, whole *F8* sequencing and RNA analysis are now implemented for genetic analysis in HA showing that deep intronic variations are a recurrent molecular mechanism causing hemophilia-A.^10–12^

A GWAS on >46,000 normal individuals has found 11 susceptibility loci for plasma VWF levels.^13^ The majority of VWF loci fall into 3 categories: genes that modify either VWF glycosylation, secretion, or clearance. Gene panels have been established by different consortia, which include the commonly affected genes. About 50% of patients with inherited thrombocytopenias and about 25% of patients with inherited platelet function defects can be diagnosed by NGS. Serious concerns have been raised regarding the genetic diagnosis of particular disorders which are rather mild in terms of bleeding manifestations. However, these are associated with an increased risk of developing hematologic malignancies. Careful and detailed discussions between the treating physician and the patient should precede testing.

### Proposed research for the roadmap

Susceptibility loci were discovered when large national biobanks with genomics data (GWAS or NGS) and VTE or bleeding phenotypes (including patients with VWD type 1 and HA with no mutations on *VWF* and *F8* loci) from electronic health files are combined. The identification of structural variants remained challenging and the development of single-molecule long-read sequencing technologies should improve the diagnosis rate. Moreover, it has been proposed recently that miRNA dysregulation is a likely cause of the disease in HA patients. It is, however, very likely, that in many patients, the reduced activity of coagulation factors are combinations of dysfunction of the protein and reduced expression of the gene. This hypothesis is bolstered by findings that different patients with the same missense *F8* mutation may exhibit mild, moderate, and severe forms of the disease based on FVIII activity. MiRNAs, whose principal biological function is fine-tuning of gene expression, are likely candidates for maintaining the balance between preventing both, excessive bleeding and clots that could lead to thrombosis. Specific goals are as follows:

- Conduct coordinated functional experiments on recent genomic discoveries to characterize functions of new genes linked to VTE and VWF and to identify causal variations.
- Deploy agnostic approaches including GWAS to identify genetic risk factors specific for recurrence and high-risk populations.
- Perform a systematic screening of known VTE genes or even the whole exome/genome, instead of the limited thrombophilia screening in well selected patients with a strong family history of VTE and a personal unprovoked event at young age.

### Anticipated impact of the research

Until recently, pathophysiology of VTE was only based on the original model of coagulation. All current pharmacological agents to treat and prevent VTE, including warfarin and the newer direct anticoagulant drugs disrupt the coagulation pathway. This interference with hemostasis increases the risk of potently lethal bleeding events. Accumulating data has highlighted mechanisms independent of coagulation cascades on clotting formation suggesting the possibility of developing innovative treatment with less side effects.

For bleeding disorders, new disease-causing variants will be found using new molecular approaches described earlier. This will complete genetic counseling for these patients, allowing genetic family investigation and prenatal diagnosis.

Despite its power in improving our understanding of the biology of human diseases, genetics is not the panacea and other molecular determinants deserve to be more intensively investigated in relation to VTE and bleeding risk. These include metabolites, epigenetic marks, noncoding RNAs, and others. Recent high-throughput technologies now allow one to profile biosamples (serum, plasma, and whole blood) for such molecular phenotypes. Some isolated initiatives have been launched to identify novel players contributing to VTE risk,^14–17^ with very promising results. These efforts should be pursued, both, for thrombosis and bleeding at the European level.

## Coagulation activation in the context of immune reactions

### Introduction

**Figure 1.**
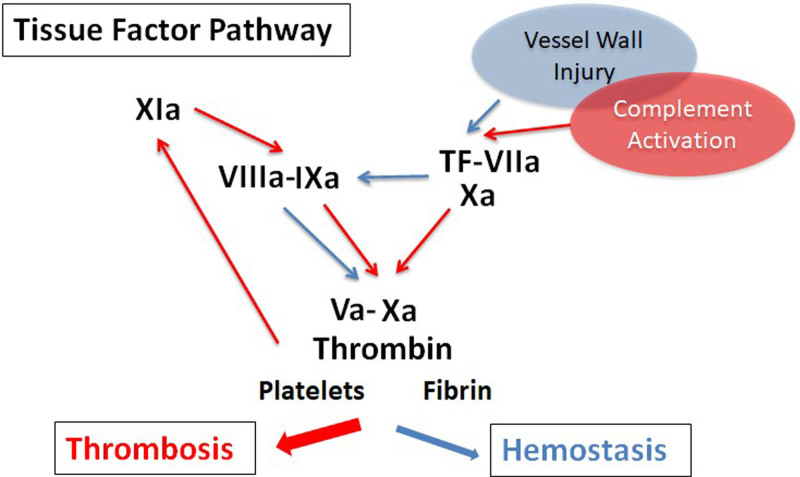
**Coagulation activation by the TF pathway with proposed connections to thrombosis (red arrows) and hemostasis (blue arrows).** TF = tissue factor.

Activation of the plasmatic coagulation cascade generates the key enzyme thrombin that is pivotal for converting the soluble plasma protein fibrinogen to fibrin and the activation of blood platelets in hemostasis and thrombosis (Figure 1). Blood coagulation frequently accompanies immune and inflammatory reactions. Recent years have uncovered several fundamental mechanisms that connect the hemostatic system with immunity. Multicellular interactions involving coagulation, leukocytes, platelets, and the endothelium are central mechanisms of antibacterial host defense, causing immunothrombosis in cardiovascular diseases and severe bacterial and viral infections.^18^ In addition, activation of the extrinsic or tissue factor (TF) and coagulation pathways play a pivotal role not only in the hemostatic response^19^ but also directly in inflammation as well as innate and adaptive immunity through coagulation proteases which signal by cleaving protease activated receptors (PAR).^20^ Interactions between the coagulation and complement cascades and contributions of these systems to immune reactions and autoimmunity are emerging areas for future research with high priority.

### European research contributions

European scientists were major contributors to recent conceptual advances in our understanding of new molecular connections regulating thrombus formation and resolution and how new antithrombotic targets are linked to vascular inflammation^21^ and chronic inflammatory processes.^22^ In this context, thrombin binding to the platelet glycoprotein Ib receptor has emerged as a potential target for ameliorating chronic liver inflammation in nonalcoholic steatohepatitis^22^ and as the activator of coagulation FXI in vascular inflammation leading to hypertension.^21^ Our understanding of coagulation reactions is still evolving and the direct activation of the intrinsic antihemophilic factors VIII and IX have been identified as an immediate function of the TF coagulation initiation complex that occurs before shutdown of this complex by TF pathway inhibitor.^19^ This and other results^23^ are beginning to define pathways that more selectively contribute to hemostasis versus thrombosis. In addition, FXIa is an attractive target and is currently being evaluated clinically for prophylactic antithrombotic therapy (Figure 1).

The antimicrobial host defense complement system has multiple connections with coagulation, and genetic mutations in key regulatory factors of complement can cause chronic vascular and hematological diseases. Complement factors 3 and 5 are crucial for venous thrombosis by acting as the trigger for rapid activation of platelets as well as TF on monocytes leading to fibrin formation.^24^ Complement is a major amplifier for disseminated intravascular coagulation in sepsis and recent data show that antiphospholipid antibodies not only require coagulation-complement crosstalk to induce thrombosis but also to elicit pathogenic signaling as the underlying cause of the autoimmune antiphospholipid syndrome.^25^ Complementary research has identified pathophysiological relevant activators of coagulation, including DNA and RNA released in the context of cell damage and polyphosphates derived from platelets and microbial pathogens. It is remarkable that autoimmune signaling of antiphospholipid antibodies utilize extracellular RNA for pathogenic effects, and the development of autoimmunity in mouse models^26^ points to a plethora of reciprocal interactions of immunity with coagulation.

### Proposed research for the roadmap

Coagulation research has generated novel diagnostic technologies, preclinical animal models, and translational approaches to develop a profound understanding of the regulation of coagulation initiation in specific vascular and extravascular locations. This productive path should be continued with a special focus on understanding the common and discriminating regulatory mechanisms of coagulation in thrombosis versus hemostasis. Immune mechanisms in thrombosis, the interaction of coagulation with the immune system, and the direct role of coagulation PAR signaling in innate and adaptive immune responses^27^ are specific research areas with a high likelihood of return. Ultimately, this research will improve anticoagulant therapy not only for thromboprophylaxis but also in the context of thromboinflammatory and aging-associated diseases. Research should also address the similarities and differences of multicellular interactions under different flow conditions and the implications of these mechanisms for more specific targeting of coagulation to prevent thrombosis in the venous and arterial system. The following are the specific topics of continuing interest:

- Triggers, modulators, and regulators of intrinsic and extrinsic coagulation activation in the venous and arterial circulation.
- Contributions of the coagulation system to the complex multicellular interactions in intravascular and extravascular pathological conditions.
- Thromboinflammatory circuits contributing to vascular dysfunction, chronic inflammatory, and autoimmune diseases.
- Environmental, metabolic, age, and gender effects on the reciprocal interactions of the hematopoietic and the coagulation systems.

### Anticipated impact of the research

These areas of research are of high significance for the rapidly evolving landscape of antithrombotic therapy and the choice of appropriate drugs for hemato-oncological and cardiovascular diseases. New insights into coagulation pathomechanisms will drive innovation toward individualized antithrombotic therapy with new anticoagulants in complex thromboinflammatory diseases.

## Infection and coagulopathy

### Introduction

Infections are often accompanied by activation of the blood coagulation system. These abnormalities range from subtle activation of hemostasis that can only be detected by sensitive markers for coagulation activation to somewhat stronger. Here, hemostatic activation may be detectable by a small decrease in platelet count and subclinical prolongation of global clotting time, to fulminant disseminated intravascular coagulation (DIC). This is also characterized by widespread simultaneous microvascular thrombosis and profuse bleeding.^28,29^

We also see ample evidence that activation of coagulation in concert with inflammatory activation can result in microvascular thrombosis thereby contributing to multiple organ failure in patients with severe infections.^30^ Infection-associated coagulopathy has shown to be an independent predictor of organ failure and mortality in patients with sepsis.^31,32^ In a consecutive series of patients with severe infectious disease, the mortality of patients with DIC was 43%, as compared with 27% in those without DIC.^33^

Infection-associated coagulopathy is most frequently associated with bacterial sepsis. Sepsis is a major health problem with an estimated 48.9 million incident cases worldwide and 11 million sepsis-related deaths in 2017, representing a fifth of all global deaths that year.^34^ Total in-hospital mortality of sepsis is around 20%, whereas severe sepsis is associated with mortality rates of 40%–50%.^35^ If sepsis is complicated by coagulopathy, mortality significantly increases. Activation of the coagulation system has also been documented for nonbacterial pathogens including viruses, protozoa (malaria), and fungi. Common viral infections such as influenza, varicella, rubella, and rubeola are rarely associated with DIC.^30^ Other viral infections can cause “hemorrhagic fever” characterized by fever, hypotension, bleeding, and renal failure. Examples are Ebola and dengue-related hemorrhagic fever.

Another example for an infection-related derangement of coagulation is COVID-19.^36^ Many patients with severe COVID-19 infections present with coagulation abnormalities that mimic other systemic coagulopathies associated with severe infections. This includes DIC or thrombotic microangiopathy but also have distinct features.^37^ The occurrence of coagulopathy in SARS-CoV-2 patients is associated with a higher risk of death.^38^ Furthermore, the relevance of coagulation abnormalities associated with COVID-19 becoming increasingly clear as a substantial proportion of patients with severe COVID-19 infections develop venous and arterial thromboembolic complications.^39,40^ It has even been hypothesized that in a subgroup of patients a sudden deterioration of respiratory function might be related to pulmonary embolism, which compromises oxygen exchange and may cause hemodynamic instability.

A variety of relevant mechanisms contributing to the derangement of coagulation in infections have been elucidated. Initiation and propagation of coagulation with concurrent impairment of physiological anticoagulant pathways and a deficit of endogenous fibrinolysis will result in systemic inflammatory activation resulting in platelet activation and fibrin deposition.^41^ In addition, recent work indicates that intravascular webs (“neutrophil-extracellular traps”) composed of denatured DNA from destructed cells and entangling neutrophils, platelets, fibrin, and cationic proteins, such as histones, may play a crucial role in the development of thrombus deposition.^42^ Infection- and inflammation-induced endothelial cell perturbation and injury may cause increased platelet-vessel wall interaction, due to the release of high molecular weight multimers of von Willebrand factor, insufficiently cleaved by deficient ADAMTS13 resulting in thrombotic microangiopathy in the microvasculature.^43^ COVID-19 and other coronavirus infections are clear examples of direct viral infection of endothelial cells.^44^

### European research contributions

The extensive unraveling of the interaction between inflammation and coagulation has for an important part taken place in European research laboratories. In particular, many of the experimental infectious disease models have been extensively employed to dissect the distinct pathways leading to coagulation activation in infectious disease have been made operational by European research groups.^41^

In addition, European investigators have been leading or importantly contributing to clinical studies evaluating novel interventions aiming at the retration of specific components or pathways in the coagulopathy associated with infections. Large randomized controlled trials (RCTs) on cytokine treatment or natural anticoagulants have been initiated by European groups. European research groups initially from Italy and Spain were the first to precisely describe the COVID-19 coagulopathy, its relationship to increase incidence and venous thromboembolic disease in these patients and have proposed potential management strategies.

### Proposed research for the roadmap

Although insights into the mechanisms underlying the development of coagulopathy in infectious disease have improved in recent decades, still important questions remain.^45^ Substantial knowledge has accumulated on individual pathways and systems contributing to the infection-associated coagulopathy. A more integrated view needs to be developed focusing on the interaction of various systems that are involved in the pathogenesis. This includes endothelial cells, platelets, plasma, complement factors, inflammatory cells, and mediators. For instance, it is likely that activation of coagulation in DIC occurs at the surface of the endothelium that interacts with inflammatory cells and mediators, and we must gain more knowledge on the exact interactions between these components at the surface in vivo. Since most of our current insights are based on in vitro observations (for example, by using cultured cells and isolated molecules), this might lead to spurious results. Studies in experimental animals and humans are mostly based on ex vivo observations, the real challenge maybe able to more directly and incisively analyze inflammatory-driven activation of coagulation at the vascular wall surface in vivo. This approach could potentially yield new targets for improved treatment strategies for infection-related coagulopathy.^45^

#### Better treatment for coagulopathy

Current therapeutic interventions are mostly supportive and only partly effective. Although these interventions can lead to improvement of the coagulopathy or more rapid resolution of coagulation derangements, they do not affect clinically relevant outcomes such as organ dysfunction or mortality. Further refinement of supportive treatment might come from the notion that the effect of the coagulopathy in infections might vary from organ to organ.^46^ Hypothetically, it could be argued that therapy should be tailored to organs that are most affected. For example, if acute lung injury is the most prominent feature, therapy should be aimed at restoration of physiological anticoagulant pathways such as antithrombin or thrombomodulin. In severe sepsis presenting with purpura fulminans, there are ample indications that restoration of the activated protein C pathway might be most effective. By contrast, in acute renal failure, interventions aimed at the deranged platelet–vessel wall interaction (eg, restoring the levels of ADAMTS13) can be most helpful.^43^ Another example is the COVID-19-related coagulopathy where a better understanding of the underlying pathogenesis is important to come to better preventive and treatment strategies to manage thromboembolic complications and to improve the outcome.

#### Risk management and diagnosis

Management of infection-associated coagulopathy might also benefit from improvement in early patient identification and risk stratification. Although the diagnosis of coagulation defects has been substantialy improved and facilitated after the introduction of diagnostic scoring algorithms and new techniques, these systems are particularly effective in establishing overt caogulopathy and less sensitive and specific for coagulation derangement in its early stages.^47^ In addition, tests that can be used to assess endothelial cell perturbation in combination with early-stage systemic coagulopathy would be helpful to identify patients at high risk of developing uncontrolled coagulopathies and would facilitate early (and thereby potentially more effective) treatment.

#### Genetics

Genetic variation between patients might be important for the vulnerability of the development of coagulation abnormalities and the severity of this condition.^48^ For instance, genetic mutations and polymorphisms have been shown to affect coagulation and fibrinolysis in DIC. Mice with targeted disruption of one allele of the gene encoding protein C causes heterozygous protein C deficiency, exhibited a more severe DIC and the associated inflammatory response.^49^ In addition, factor-V Leiden heterozygosity has been associated with both, incidence and outcome of DIC in sepsis.^50^ Furthermore, a functional genetic variation in *SERPINE1* coding for PAI1—the 4G/5G polymorphism—not only influences the plasma levels of PAI-1 but has also been linked to clinical outcome of meningococcal sepsis and coagulopathy.^51^ More insight into the genetic variations that influences the host response to conditions underlying coagulation abnormalities might be helpful for identifying patients who are more susceptible to this complication and for individually tailoring therapy to the most vulnerable patients.

### Anticipated impact of the research

Infection-induced coagulopathy and its resulting cinical complications, both thromboembolic disease and bleeding, have an important impact on the outcome for patients with severe infections. A more thorough and integral understanding of the underlying pathways may lead to better points of impact for supportive treatment strategies that might have a beneficial effect on clinical course, organ dysfunction, and, ultimately, mortality.

Once the safety and efficacy of new adjunctive treatment strategies aimed at the coagulation system has been established, the management of critically ill patients with severe infections can considerably improve. This is potentially leading to shorter duration of intensive treatment and a reduction in transfusion requirements.

A more individual approach based on patient characteristics maybe helpful in more precise patient selection for specific treatment strategies, which may further increase the effectiveness and safety of treatment targeting the coagulation system in patients with severe infections.

## Venous thromboembolism

### Introduction

VTE comprising deep vein thrombosis and its sequelae pulmonary embolism occurs in 1–2/1000 people per year and is one of the leading causes of death in Europe.^3^ Importantly, VTE is a number one preventable disease as antithrombotic regimens are highly effective.

### European research contributions

European researchers were masterminds behind the development of novel direct oral anticoagulants (DOACs) and their introduction into clinical practice. DOACs are effective and safe in preventing thrombotic events in patients with VTE including those with cancer, atrial fibrillation, or arteriosclerotic disease. Furthermore, pivotal insights have been gained in linking the mechanisms underlying thrombosis, inflammation, and innate immunity.^52^ European researchers are also leading in unravelling the mechanisms initiating coagulation in VTE. Emerging data suggested that, in addition to TF, the contact system plays a part in the initiation of coagulation in VTE.^53^

Animal models indicated that platelets likely contribute to VTE, which explains why aspirin is effective for its prevention albeit less than anticoagulants.^54,55^

### Proposed research for roadmap

Following up on these recent advances, future research on VTE should focus on the following:

1. Improving antithrombotic treatments. DOACs have transformed the prevention and treatment of VTE, but bleeding remains the major complication with a 3-month rate of major bleeding of about 3.3% in clinical practice and even higher in older patients and in those with chronic kidney disease or cancer.^56^ Therefore, there remains a need for safer anticoagulants. Preliminary favorable results of clinical trials are already available for several FXIa inhibitors that may provide new opportunities to reduce the burden of VTE and bleeding.
2. Improving our knowledge on the thrombotic risk and underlying pathomechanisms in specific subgroups including patients with cancer, phospholipid antibodies, and kidney disease also in elderly patients, children, infants, or pregnant women.
3. Enabling the differentiation between high- and low-risk patients not only for VTE but also for bleeding and integrating the 2 on the basis of clinical, genetic, and molecular evidence. This will lead to personalized systems for assessing the thrombosis and bleeding risks which allows individualized, targeted and, thus, safer antithrombotic concepts.
4. Developing “learning healthcare systems.” Relying almost exclusively on RCTs will not allow for translating the current pace of research progress into new and better treatment. It will also enable decision makers to navigate complex scenarios in the future, including regulatory, financing, or patient-level decisions. Real-world data (RWD) based on electronic health records and other routinely collected data are readily available particularly in VTE patients. A “learning healthcare system” based on electronic health records and other routinely collected data could harness the full potential of RWD to complement evidence based on RCT.^57^
5. Advancing assay systems reflecting venous thrombus formation in vivo. Currently, pathogenesis concepts of the VTE rely on in vitro and animal models as exploration of thrombus formation is not feasible in man. Novel systems as close as possible to human in vivo conditions that integrate all important determinants of thrombus formation, such as blood cells, cell fragments, coagulation factors, endothelium, and flow, are urgently needed to improve our understanding of VTE pathogenesis and the specific role of the individual components.
6. Consolidating the novel concepts of inflammation, thrombosis, and innate immunity with a particular focus on distinguishing treatable proinflammatory from host-protective pathways.

### Anticipated impact of the research

The large number of people suffering from VTE remains a serious challenge to European health care systems. The research proposed in this document will reduce the disease burden related to VTE occurrence and antithrombotic induced bleeding. After the vast achievements that have already been made in the general population regarding prevention of first and recurrent VTE, a special focus should be placed on patient groups with cancer, phospholipid antibodies, kidney disease also in elderly patients, children, infants, or pregnant women in whom the balance between the risk of thrombosis and bleeding is particularly vulnerable. Electronic health records and RWD are an important but underused resource. Analytics methods will improve effectiveness and efficiency of the development and use chain, including research and development, regulatory decision-making, health technology assessment, pricing and reimbursement decisions, and treatment. Further unraveling the molecular pathways and effector functions of the interplay of innate and adaptive immunity, coagulation and platelets in inflammation and infection, and the development of thrombotic complications will offer new potential therapeutic approaches.

Only a concerted action bringing together basic scientists and clinically orientated researchers will eventually be successful at achieving such ambitious goals.

## Bleeding disorders

### Introduction

Clotting factors deficiency and platelet abnormalities are hereditary bleeding disorders that lead to recurrent hemorrhagic events, both, spontaneous and traumatic. Disabling conditions with a reduction in quality of life are observed in all age groups of people affected by these disorders.

Every year, demographic data in people with hemophilia, VWD, rare factor deficiencies, and inherited platelet disorders worldwide are updated by the World Federation of Hemophilia (WFH) and published in the “Annual Global Survey.” In 2020, people affected with rare bleeding disorders are 324,648 located across 125 countries.^58^ In recent years, knowledge on bleeding disorders has significantly improved, leading to a dramatically improvement in both, diagnostic and clinical management, particularly in hemophilia patients. The implementation of new technologies such as NGS or droplet digital PCR system has modernized and shortened the detection time of molecular characterization in patients with bleeding disorders in female carriers of these diseases. Prenatal diagnosis has dramatically changed over the last 2 decades with the introduction of noninvasive methods (NIPT) replacing invasive procedures. In addition, preimplantation genetic testing (PGT) has become an important reproductive tool to prevent genetic diseases.

Today, patients affected with bleeding disorders such as hemophilia have a wide spectrum of treatment options including novel extended half-life products, nonreplacement therapies, and gene therapy. The shift from standard Factor VIII and IX concentrates on novel products has rendered hemophilia treatment less distressing with an improvement of patients‘ quality of life. In addition, nonreplacement therapies such as fitusiran or anti-TFPI antibodies are now in phase 3 clinical trials which could probably be used not only in hemophilia but also in some other rare bleeding disorders. Overall, gene therapy is becoming a promising therapeutic option for both, hemophilia A and B, having demonstrated significant expression and durability in clinical trials.

### European research contributions

In the last few years, a large number of real-life data have shown the validity of extended half-life factor VIII and IX products as well as nonreplacement therapy such as Emicizumab, a humanized-bispecific antibody that mimics FVIII function in the coagulation cascade. These therapies have introduced a significant benefit into the patients’ daily lives by simplifying prophylaxis regimen using subcutaneous drug administration with fewer infusions. However, some very rare clotting disorders such as FV and FII deficiency as well as platelet abnormalities are not associated with specific drugs. Consequently, there is a need to accelerate the production of generic biosimilar drugs in the field of bleeding disorders.

The collection of long-term safety data planned at the European level for newly approved drugs for gene therapy is also a crucial point to address. A currently active multinational program for monitoring safety of treatment is the European Haemophilia Safety Surveillance (EUHASS), with 96 participating centers distributed to 30 European countries.^59,60^ The ISTH Scientific and Standardization Committee on Factor VIII, Factor IX, and other rare coagulation disorders have also published a minimal dataset necessary for postregistration surveillance. The European Medicines Agency (EMA) has recommended a harmonization of systems for collecting data on safety and efficacy of novel drugs at the European level.^61^

Recent data have proven that gene therapy is effective and safe for the treatment of patients with both, hemophilia A and B. Ten gene therapy studies using adeno-associated virus (AAV) vectors for hemophilia A and 6 for hemophilia B are currently in progress. Three of these trials are in phase 3 for hemophilia A and 2 for hemophilia B. These trials, rather frequently reported the elevation of serum transaminases, sometimes associated with a transitory decline in plasma factor levels.

Suspension by the US Food and Drug Administration of the hemophilia B gene therapy program (AMT-061; ethranacogene dezaparvovec) due to a serious adverse event such as hepatocellular carcinoma in a patient 1 year after treatment during phase 3 has recently been lifted.^62^ Comprehensive investigation showed that AMT-061 (ethranacogene dezaparvovec) is very unlikely to have contributed to the carcinoma in the patient. The vector toxicity is linked to the ability to integrate into the host genome, but AAVs are not considered as integrating vectors existing mainly in the nucleus in episomal form, although random integration events occur but at very low frequency. A recent study in animal models has shown that fragments of the vector sequence were integrated into the genome of a dog model without any association with evidence of malignancy. Postmarketing surveillance in gene therapy, therefore, is a crucial issue that needs capturing unexpected or rare events that occur in patients receiving such therapy.

Thanks to international collaboration with WFH, ISTH, the European Haemophilia Consortium (EHC), the US National Hemophilia Foundation, the American Thrombosis and Hemostasis Network as well as industry gene therapy partners and Regulatory liaisons, a world Gene Therapy Registry (WFH) has been formulated with the aim of providing a robust and valid data collection system available to all healthcare caregivers who follow patients treated with gene therapy.^63^ Most of the scientific progress and innovation in therapy has been obtained from the pharmaceutical research laboratories and not from academia. Therefore, dedicated funding to academic institutions in the coming years will be needed to guide research in the field of rare bleeding disorders.

### Proposed research for the roadmap

The following research agenda needs to be addressed:

1. Clinical management of patients by specialized care centers (European Haemophilia Comprehensive Care Centres [EHCCCs]). Specialized care centers should prepare specific standardized health procedures for the management of hemophilic patients (with and without inhibitors) using new nonreplacement drugs, with or without the addition of other haemostatic products during breakthrough bleeding or at the time of surgery. A specific accreditation model for hub/spoke or EHCCC care is needed. It is important that patient management also occurs in collaboration with clinical researchers to better understand the new therapeutic strategies. The involvement of expert immunologists and hepatologists would be most important to understand any side effects of gene therapy trials, such as transaminitis, use of steroid therapy, its duration and other issues. European Association for Haemophilia and Allied Disorders and EHC are promoting the hub model for the treatment and monitoring of patients with hemophilia and rare bleeding disorders treated with gene therapy.
2. There is an urgent need to better understand the mechanisms of action for novel nonreplacement drugs such as bispecific antibodies, anti-TFPI, fitusiran (siRNA), and others in patients with hemophilia. These new drugs act by decreasing natural anticoagulants, determining an imbalance in the natural clotting process. The exact procoagulant activity and also the hemostatic potency of these drugs are not completely understood. Therefore, the management of patients in specific challenging situations such as surgery, the elderly, acute bleeding, and others should be carefully considered.
3. Data monitoring on novel therapies by specialized laboratories and qualified staff are required. Discrepancies between FVIII and FIX assays have been described for new extended half-life products including emicizumab and also other gene therapy trials. In view of the aforementioned, the specialized EHCCC laboratory should guarantee the application of the most appropriate tests for each clinical situation and a correct interpretation of the results to ensure an accurate diagnosis and optimal and safe therapeutic management of patients.
4. The future direction of hemophilia treatment is gene therapy, although there are still some critical points needing to be addressed when determining the cause of liver toxicity triggering the increase in liver enzymes, and overcoming liver transduction inhibition due to pre-existing anti-AAV antibodies against the capsid. To solve this problem, new viral vector manufacturing processes are emerging, as well as the selection of other AAV serotypes. However, more collaboration between academia and manufacturers is required to solve major unmet medical needs.
5. Neutralizing antibodies against FVIII administered at the early stage of life is still up to 30% of patients with severe hemophilia even with novel extended half-life products.^64^ An answer has been given in understanding which type of product could most likely cause the inhibitor development.^65^ However, the immunological mechanisms that trigger this immune response needs further investigation.
6. Long-term postmarketing monitoring of the efficacy and safety for all therapies using a patient registry for hemophilia and other bleeding disorders is required. Each European nation should have a register of patients with rare diseases and in particular with hemorrhagic disorders, to collect data on usage of products, their efficacy and side effects.
7. There is a need for legal provision with specific amendment to ensure that regulators accommodating new scientific developments allowing scientific collaboration between academic and pharmaceutical centers.
8. Unnecessary multiplication of procedures should be avoided to render drug registration at European level more efficient and less burdensome.
9. A revised system of incentives to boost the research and innovation in new therapeutic areas is required (very rare disorders, as FV, FII, and inhibitor development).

### Anticipated impact of the research

The widespread use of new drugs has led to a significant change in the clinical management of patients with bleeding disorders. Therefore, it is necessary to stress that the treatment of patients should be managed by specialized European treatment centers organized as a model very similar to the hub and spoke system, ensuring strategic organization and leading to the provision of optimal levels of treatment and care.

In addition, laboratories must adopt appropriate assays for postinfusion measurement of new drugs to have an appropriate correct monitoring of treatment. Collaboration between experts in thrombotic and bleeding disorders is fundamental for scientific improvement.

Dedicated funding is needed for an accurate research program on the side effects of novel nonreplacement therapies in hemophilia.

**Summary box: Main research & policy priorities**

1. Developing high-throughput technologies for profiling biosamples for molecular determinants (genetics, metabolites, epigenetic marks, and noncoding RNAs) to improve understanding of pathophysiology of thrombotic and bleeding disorders and hence treatment
2. Improving integral understanding of underlying pathways of Infection-induced coagulopathy enabling precise patient selection to offer specific treatment strategies which will increase the effectiveness and safety of treatment targeting the coagulation system in patients with severe infections.
3. Unraveling the thromboinflammatory circuits contributing to vascular dysfunction, chronic inflammatory, and autoimmune diseases.
4. Implementation of learning healthcare systems in venous thromboembolic disease based on real-world data and electronic health records to improve effectiveness and efficiency of the development and use chain, including research and development, regulatory decision-making, health technology assessment, pricing and reimbursement decisions, and treatment.
5. Establishing specialized European treatment centers and laboratories to ensure strategic organization and provision of treatment and care, appropriate assays for postinfusion measurement of new drugs and to evaluate side effects of novel nonreplacement therapies in hemophilia.

## Disclosures

SE received royalties of payments from Bayer, BMS, Boehringer-Ingelheim, Daiichi-Sankyo, CSL-Behring, Takeda, and Pfizer. MC is an Advisory board member in Astra Zeneca. AR received research grant from CSL Behring, LFB, and Roche/Chugai; a clinical trials investigator for Biomarin, Bioveratin, CSL Behring, Shire/Takeda, and Sobi; and an Advisory board member for LFB, Octapharma, Roche, and Sobi. WR is a consultant for ARCA Biopharma and ICONIC Therapeutics; received research grants from ICONIC andARCA Biopharma; and ownership interest for MeruVasimmune. ML is in Occasional Advisory Boards of Lilly, Novo Nordisk, and Asahi Kasei. JAP received royalties of payments from Stago, Octapharma, and CSL Behring. FP participated in educational meetings and advisory boards for Sanofi, Roche, Sobi, and Takeda. All the other authors have no conflicts of interest to disclose.
